# Professor Faraday on the Cohesive Force of Water

**Published:** 1846-08

**Authors:** 


*Professor Faraday on the Cohesive Force of Water.*—This was the last evening meeting, for the season, of the members of the Royal Institution, and on this occasion Prof. Faraday gave an interesting lecture on the "Cohesive Force of Water." The lecture was so strictly experimental, that we can do little more than give the heads of some of the most striking illustrations which were brought forward.

The Professor first adverted to the three conditions of water, as ice, water and steam. Bodies which appeared perfectly liquid were, in reality often in a transition state, *i. e.* ready to take on the solid form from the slightest mechanical disturbance in their particles. This was well known to be the case with a saturated solution of sulphate of soda which might be kept for a year in the state of a perfect liquid like water, but would in an instant, from any mechanical cause, such as mere agitation or the addition of any solid, become converted to a crystalline mass. There was a somewhat analogous condition with regard to liquids, and vapours discovered by M. Cagniard de la Tour, and known under his name. At a certain temperature a liquid under sufficient pressure becomes a clear, transparent vapour or gas, having the same bulk as the liquid.

Water, as a liquid, was supposed at one time to be destitute of all cohesive force, but the slightest attention to its properties proves that this is an error. When solids are immersed in water, a certain portion of the liquid adheres to them, and this is called adhesion. This, however, only refers to the mere film of liquid on the surface of the solid; and as a drop or globule can be accumulated, it is obvious that the particles of water must have among themselves, a strong force of cohesion, since they are able to resist the power of gravitation. Various experiments have been devised for the illustrations of this property. Among others the familiar one of balancing a smooth glass plate, having a known area, at the end of a scale beam, bringing this plate in contact with a surface of water, and then observing the weight required in the opposite scale to raise it. It will be found to be much more than an equivalent to the weight of the glass plate, provided care has been taken by covering the surface of the glass with a thin layer of soap, to ensure perfect contact. Laplace thought that the weight required to lift the plate was an index of the force of cohesion between water and glass, but this is not the true explanation. The weight added to the opposite scale really indicates the cohesive force existing among the particles of water themselves; for before the plate is detached from the surface, it will be seen that a mass of water is actually raised up, forming a well-marked curve with the surface of the liquid. Some ounces are thus required to lift a plate exposing an area of twenty-five square inches.

The Professor next adverted to some remarkable experiments performed by M. Donné, a Belgian plilosopher, and M. Henry on the cohesion of water. Donné found that if water were enclosed in a tube bent at right angles, and hermetically sealed on the principle of the water hammer, provided there be no air in a free state, and one leg of the tube is completely filled with water, it might be inverted, *i. e.* turned with the angle downwards, without the water finding its level on the usual hydrostatic law. This experiment was shown: and it was thus rendered apparent, that there was a great force of cohesion in water, since a column of twelve or fourteen inches in height, and three quarters of an inch in diameter, was thus sustained against gravity. When, however, the tube was gently shaken, a bubble of air, hitherto dissolved in the water, was dislodged by the concussion, rose to the surface of the water, and this immediately fell until the hydrostatic equilibrium was established. When another tube containing water free from air was taken, it was found impossible to dislodge the column of water, or to produce the effect of the water-hammer, without incurring the risk of breaking the tube. M. Henry had calculated from certain experiments that the cohesive force of water was equal to some hundreds of pounds pressure. He also made the remarkable discovery that the boiling point of water, entirely deprived of air by shaking it in the manner above described, was no less than 275°; and that when this temperature was reached, a large body of steam was suddenly generated with explosive force, much in the same way in which the boiling of concentrated sulphuric acid is observed to take place. The boiling point of water is thus raised to a temperature equivalent to a pressure of three atmospheres, or forty-five pounds on the square inch. This result, therefore, enables us to form an idea of the power whereby the particles of water are held together; for it is undoubtedly the force of cohesion among them which enables them to resist the action of heat until so high a temperature has been reached. Were it not for the air contained in it, water would not boil at 212°; and the operation would be attended with considerable danger, since, when deprived of air, the boiling of this liquid is accompanied by a violent explosion.

Water is repelled by the surfaces of numerous solids, and in this fact we have an additional illustration of the great cohesive force among its particles; for the liquid invariably settles in globular masses or acquires a convex surface. The dew on the leaf was a well-known instance; but there is another remarkable illustration of this property, in fact that the meshes of fine wire gauze are not traversable by water, notwithstanding its perfect liquidity. This was proved by pouring water into a small cylindrical vessel made of copper-wire-gauze. The water remained in it without flowing out, just as ifthe sides were formed of solid matter, instead of being perforated with an infinite number of holes. It was not until the vessel was shaken that a communication could be established with the outside, and the water flowed away. A strong cohesion might be set up between water and metal by covering the top of an open jar with fine wire gauze and moistening the surface. A thin film of water was locked up in each mesh of the gauze ; and so strong was the cohesion, not merely of the water to the metallic copper, but of the particles of water themselves, that atmospheric pressure was resisted, and the vessel plunged into water and raised, was now capable of supporting a column weighing several pounds. Professors Faraday then showed that water would not traverse fine wire-guaze, so long as this was at a red heat or near it. A cup of very fine wire-gauze was heated to redness, and some water poured on. It rolled over the surface like a rounded mass of quicksilver, so long as the heat was maintained ; but when the lamp was withdrawn, and the wire allowed to cool, the water speedily traversed the meshes, producing an abundance of steam.

It is an old and well-known fact, that water will lie upon red hot sur faces without boiling or producing any perceptible quantity of vapour. The same law applies to all liquids. Water was poured on a red hot vessel, and it remained like quicksilver in constant motion, but without producing visible steam. In all these cases there was no immediate contact between the liquid and red hot metal. Contact was entirely prevented by the interposition of a thin film of very dry vapour : and it had been found, singularly enough, that the liquid while on the red hot metal, and not in contact, had a temperature several degrees below its boiling point. Thus water had a temperature of only 205° ; and although it underwent rapid diminution by passing off in vapour, yet this vapour, unlike ordinary steam, was perfecly invisible, owing to its being intensely dried, and probably passing at once into an atmosphere, strongly heated by radiation from the heated vessel. When the heat was withdrawn, and the metallic vessel had cooled to a certain degree, there was immediate contact between it and the liquid ; and the greater part of the latter was suddenly dissipated in steam. This was well known to be one cause of the explosions of steam-boilers. A similar experiment was then performed with ether, which is equally repelled by red hot metals ; the vapour, however, became easily ignited : but the fact of non-contact was beautifully illustrated not merely by the sight of the liquid ether rolling like mercury within the interior of the flame, but by the sudden evolution of a much larger quantity of ethereal vapour, manifested by an increase of the flame, when the vessel was allowed to cool, so as to establish contact of the liquid with the metal.

M. Boutigny has called this the spheroidal state of bodies. It is a most singular condition of matter, and the property leads to some very remarkable and unexpected results. Thus, with regard to iodine, this is well known to be a solid, giving off at a gentle heat richly coloured violet vapours. If iodine be thrown on red hot metal (platina) it melts and forms a spheroidal mass, giving off a small quantity of vapour. It has the appearance of a black liquid; and there is evidently no contact. When the vessel is allowed to cool, contact takes place, and this was indicated by the sudden burst of a large quantity of vapour of iodine from the surface of the metal.

By taking advantage of this principle M. Boutigny succeeded in freezing water in a red hot crucible, an experiment which excited considerable attention at the last meeting of the British Association. Professor Faraday then performed the experiment by making a platina crucible nearly red hot, and pouring into this, anhydrous sulphurous acid, and immediately afterwards an equal quantity of water. There was no contact between the liquid sulphurous acid and the hot metal. The acid boils at 14° F. and the evaporation is so rapid, that it cools the water poured upon it to a degree considerably below the freezing point, so that it became instantaneously a mass of ice. The lump of ice was thrown out on the table. The ice thus formed is in fact a solid hydrated compound of sulphurous acid and water, but the same effect is obtained by plunging a tube containing water into the acid,—only it requires a longer time for the freezing, and the vapour of sulphurous acid is highly suffocating. Caustic potash poured over the table tended to fix the acid and prevent the diffusion of the vapour.

It is worthy of remark, that the temperature of all liquids in the spheroidal state is a little below that at which they boil; and they pass into the spheroidal state at a temperature which is low in proportion to the lowness of their boiling points. Thus ether becomes spheroidal on metal, when only moderately heated, and retains this condition for a considerable period; while water requires a much higher temperature in the metal, and soon wets it by adhesion. Hence in freezing water in anhydrous sulphurous acid, it is not necessary to heat the metal very intensely; because this liquid assumes the spheroidal state at a comparatively low degree of heat.

In carrying out Boutigny's experiments, Professor Faraday ascertained that even mercury might be frozen under these circumstances; although the degree of cold required for this purpose is well known to be 72° below the freezing point of water. A platina crucible was made hot, and several lumps of solid carbonic acid were put into it. Ether was then poured upon the carbonic acid and a cold bath in a spheroidal state on the hot metal was thus procured, equal, in the air, to a temperature of 106° below zero; and under these circumstances the vapour of æther was not inflammable. A ladle containing liquid mercury was then introduced into the bath; and in a few seconds it was completely frozen to a solid hard button of metallic mercury! In this way, several portions of the liquid metal were successively frozen. This remarkable experiment, which was here for the first time performed in public, shows that by following out the principles of scientific discovery, results may be obtained which *à priori* appear absolutely impossible, and contrary to the laws of nature.—*Lon. Med. Gaz.*

